# Identification and validation of stromal-tumor microenvironment-based subtypes tightly associated with PD-1/PD-L1 immunotherapy and outcomes in patients with gastric cancer

**DOI:** 10.1186/s12935-020-01173-3

**Published:** 2020-03-24

**Authors:** Qianqian Ren, Peng Zhu, Hui Zhang, Tianhe Ye, Dehan Liu, Zhao Gong, Xiangwen Xia

**Affiliations:** 1 Department of Radiology, Union Hospital, Tongji Medical College, Huazhong University of Science and Technology, 1277 Jiefang Avenue, Wuhan, 430022 China; 2 Hubei Province Key Laboratory of Molecular Imaging, Wuhan, 430022 China; 3 Department of Hepatobiliary Surgery, Wuhan No.1 Hospital, Wuhan, 430022 China; 4 Department of Internal Medicine, Wuhan Hankou Hospital, Wuhan, 430011 China

**Keywords:** Gastric cancer, Stromal cells infiltration, Stromal score, Overall survival, PD-1/PD-L1 therapy

## Abstract

**Background:**

Immunotherapies targeting programmed cell death 1 (PD-1) and programmed death-ligand 1 (PD-L1) have been approved for gastric cancer (GC) patients. However, a large proportion of patients with T-cell-inflamed tumor microenvironment do not respond to the PD-1/PD-L1 blockade. The stromal component of the tumor microenvironment has been associated with immunotherapy. This study aims to explore the clinical significance of the non-immune cells in the tumor microenvironment and their potential as biomarkers for immunotherapy.

**Methods:**

A total of 383 patients with GC from the Cancer Genome Atlas (TCGA) cohort, 300 patients with GC from the GSE62254 cohort in Gene Expression Omnibus (GEO) were included in the study. A stromal score was generated using the ESTIMATE algorithm, and the likelihood of response to PD-1/PD-L1 immunotherapy of GC patients was predicted using the TIDE algorithm. The prognostic value of the stromal score from GC cases was evaluated by the Kaplan–Meier method and Cox regression analysis. Gene set enrichment analysis (GSEA) was also conducted.

**Results:**

The stromal score showed significant differences in different molecular subtypes and T stages. Multivariate analyses further confirmed that the stromal score was an independent indicator of overall survival (OS) in the two cohorts. The low stromal score group showed higher tumor mutation burden (TMB) and micro-satellite instability (MSI), and was more sensitive to immune checkpoint inhibitor according to the TIDE algorithm. Activation of the transforming growth factor and epithelial–mesenchymal transition were observed in the high stromal score subtype, which is associated with T-cell suppression, and may be responsible for resistance to PD-1/PD-L1 therapy. BPIFB2 was confirmed as a hub gene relevant to immunotherapy.

**Conclusion:**

The stromal score was associated with cancer progression and molecular subtypes, and may serve as a novel biomarker for predicting the prognosis and response to immunotherapy in patients with GC.

## Background

Gastric cancer (GC) is one of the most common malignancies, which has the second-highest tumor-related mortality rate around the world [1, 2]. The overall survival (OS) of GC remains relatively poor because the majority of cases are detected only at an advanced stage with extensive node invasion and distant metastasis [3]. For patients with unresectable/metastatic disease, systemic chemotherapy and targeted therapies, namely, monoclonal antibodies targeting trastuzumab (HER2) [4] and VEGFR2 [5, 6], offer a limited survival advantage. Inhibition of PD-1 and its ligand PD-L1 using an immune-checkpoint inhibitor has emerged as a promising immunotherapy [7]. For the treatment of patients with recurrent, locally advanced, or metastatic gastric or gastroesophageal junction cancer (GC/EGJC), whose tumors express PD-L1 by immunohistochemistry (IHC), the Food and Drug Administration (FDA) approved the use of pembrolizumab (anti-PD-1 antibody) in September 2017 [8]. However, the response rates are relatively low, which highlights the need to better elucidate the mechanisms of treatment resistance and to identify patients who will benefit the most from immunotherapy.

The degree of tumor-infiltrating T-cells in the tumor microenvironment (TME) and PD-L1 expression has been reviewed extensively elsewhere [9]. Tumors with a high level of immune infiltrates and/or an Interferon (IFN) signature indicative of a T-cell-inflamed phenotype could benefit from anti-PD-L1/PD-1 therapies [10, 11]. However, a large proportion of patients with T-cell-inflamed TME do not respond to PD-1/PD-L1 blockade [12], suggesting the presence of an additional immunosuppressive mechanism.

The stromal elements of the TME include cancer-associated fibroblasts (CAFs), myofibroblasts, myeloid cells, endothelial cells, and mesenchymal stromal cells (MSCs) [13]. MSCs and differentiated MSCs, such as CAFs, one of the most abundant and critical components of the tumor mesenchyme, influence the phenotype of the immune cells and dramatically affect tumor progression, and thus, a better understanding of the precise mechanisms involved is critical to developing more efficacious immunotherapies [14–20].

The dynamic interplay between stromal cells and the innate and adaptive immune cells involves several cellular events and physiological processes [21]. Given that stromal cell depletion therapy proceeds with caution concerning of on-target/off-tumor effects [22], the exploration of targeting common stromal-dependent molecular pathways may represent an alternative approach.

Recently, the Estimation of stromal and immune cells in malignant tumor tissues (ESTIMATE) algorithm was introduced to quantify stromal and immune components in a tumor, reflecting the tumor microenvironment [23, 24]. Several studies have shown the effectiveness of such big-data-based algorithms in different types of malignancies, including prostate cancer [25], breast cancer [26], colon cancer [27], and glioblastoma multiforme [28].

In this study, we estimated the stromal elements of TME and established a robust prognostic biomarker and predictive factor for response to immune-checkpoint inhibitors based on the gene expression profiles of GC patients. The newly found targeted genes and signaling pathways can render tumor cells more sensitive to immunotherapy.

## Materials and methods

### Database

Two cohorts of patients with GC from two independent databases, 383 patients in the TCGA-STAD database (http://cancergenome.nih.gov/) and 300 in the GSE62254 database based on GPL570 platforms (Affymetrix Human Genome U133 Plus 2.0 Array) in Gene Expression Omnibus (GEO) (http://www.ncbi.nlm.nih.gov/geo/), were included in this study. RNA-sequencing and clinical data of the TCGA-STAD patients were acquired from the TCGA database. Updated clinical data, such as gender, age, TNM stage, and survival outcome of TCGA-STAD samples, were acquired from the Genomic Data Commons. The patient cohort from the GSE62254 database was used as a validating cohort. To make the samples between two databases more comparable, RNA-sequencing data (raw values) from the TCGA database were transformed into transcripts per kilobase million (TPM) values, which is similar to microarrays in the GSE62254 database [29].

**Fig. 1:**
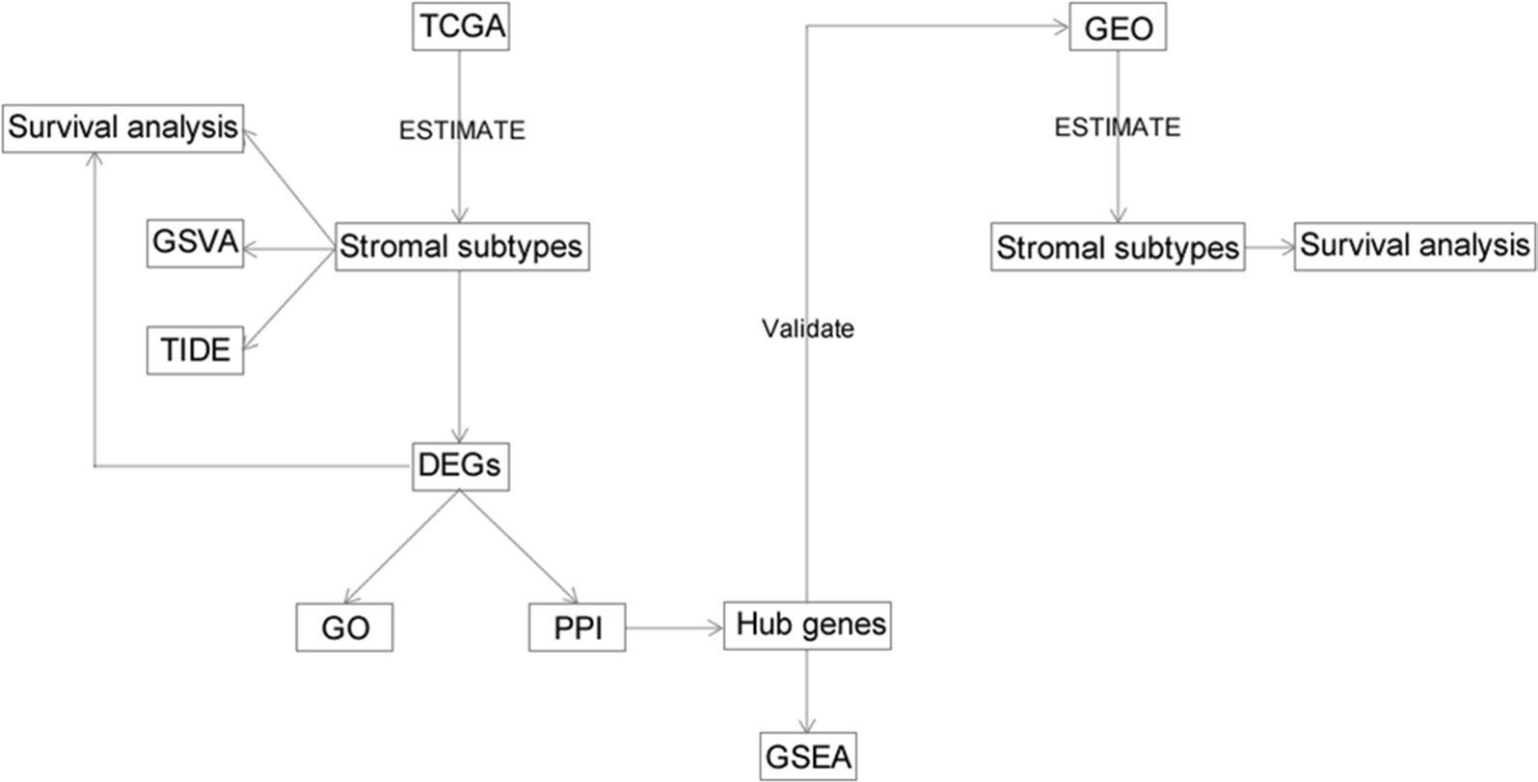
Flow chart of study design

By applying the ESTIMATE, stromal score and immune score were calculated and were used to explore the crosstalk between the stromal and immune microenvironment as well as common mechanisms of immune modulation. The TMB was calculated from comprehensive genomic profiling data. The likelihood of response to immunotherapy for each sample was predicted based on the TIDE algorithm [30]. The research design was illustrated in Fig. 1.

### Identification of deferentially expressed genes (DEGs)

Deseq2 was used for performing data analysis. SurvivalRoc package was utilized to determine the best cutoff, and DEGs were identified between high stromal score and low stromal score group using Student’s *t*-test. A *p*-value < 0.05 and absolute log2 fold change (FC) > 1 were set as the cutoff criteria to screen for significant DEGs of interest.

### Survival analysis

The Kaplan–Meier plots were generated to illustrate the relationship between patients’ OS and gene expression levels of DEGs or stromal scores. The log-rank test tested the association. Cox proportional hazards model was utilized to reveal the independent indicators related to OS.

### Functional and pathway enrichment analysis

The gene ontology (GO) functional analysis was performed using R package clusterprofiler [31]. The GO terms were identified with a strict cutoff of *p*-value < 0.05 and a false discovery rate (FDR) of less than 0.05. To explore the potential function of the hub genes in GC, we performed GSEA of the adjusted expression data for all transcripts. Gene sets were downloaded from the MSigDB database of Broad Institute (http://www.broadinstitute.org/msigdb). Enrichment p values were based on 10,000 permutations and subsequently adjusted for multiple testing using the Benjamin–Hochberg procedure to control the FDR. The enrichment scores of molecular pathways and gene expression signatures were evaluated using single-sample gene set enrichment analysis (R package GSVA).

### Construction of PPI network

We downloaded the integrated interaction information from the online database Search Tool for the Retrieval of Interacting Genes (STRING) (http://www.string-db.org/). Then we developed a protein–protein interaction (PPI) network by using Cytoscape software to further explore the relationships between DEGs at the protein level. DEGs pairs whose combined score was > 0.4 were mapped. The genes with the highest degree scores in the PPI network were selected as hub genes.

## Results

### Clinicopathological characteristics of stromal subtypes

ESTIMATE algorithm analysis showed that the stromal scores ranged from − 1957.19 to 2085.81 in the TCGA database and − 1098.03 to 5972.58 in the GEO database. Three hundred eighty-three samples from the TCGA database were separated into two subsets based on the optimal cut point of stromal score. Stromal score expression and clinicopathological factors are summarized in Table 1. Detailed clinical characteristics of the 184 patients with clinical data in the GEO database are shown in Additional file 1: Table S1.

**Table 1:** Stromal score expression and clinicopathological factors in TCGA cases

Characteristics	Stromal score		p-value
	Low (n = 192) (%)	High (n = 191) (%)	
Age (year)			0.6894
≤ 65	79 (41.1)	83 (43.5)	
> 65	112 (58.3)	106 (55.5)	
Unknown	1 (0.5)	2 (1)	
Gender			0.4945
Male	130 (67.7)	122 (63.9)	
Female	62 (32.3)	69 (36.1)	
T_stage			0.0002869
T1	18 (9.4)	2 (1)	
T2	47 (24.5)	34 (17.8)	
T3	80 (41.7)	88 (46.1)	
T4	45 (23.4)	61 (31.9)	
Unknown	2 (1)	6 (3.1)	
N_stage			0.5958
N0	60 (31.3)	51 (26.7)	
N1	55 (28.6)	51 (26.7)	
N2	38 (19.8)	36 (18.8)	
N3	32 (16.7)	42 (21.9)	
Unknown	7 (3.6)	11 (5.9)	
M_stage			1
M0	170 (88.5)	169 (88.5)	
M1	13 (6.8)	12 (6.3)	
Unknown	9 (4.7)	10 (5.2)	
Pathologic stage			0.3686
I + II	89 (46.4)	77 (40.3)	
III + IV	97 (50.5)	104 (54.5)	
Unknown	6 (3.1)	10 (5.2)	
Molecular_subtype			1.36E−06
CIN	128 (66.7)	95 (49.7)	
EBV	13 (6.8)	17 (8.9)	
GS	8 (4.2)	42 (22.0)	
HM-SNV	5 (2.6)	2 (1)	
MSI	38 (19.8)	35 (18.3)	

### The stromal score is involved in outcomes of GC subtypes and is associated with tumor progression

**Fig. 2:**
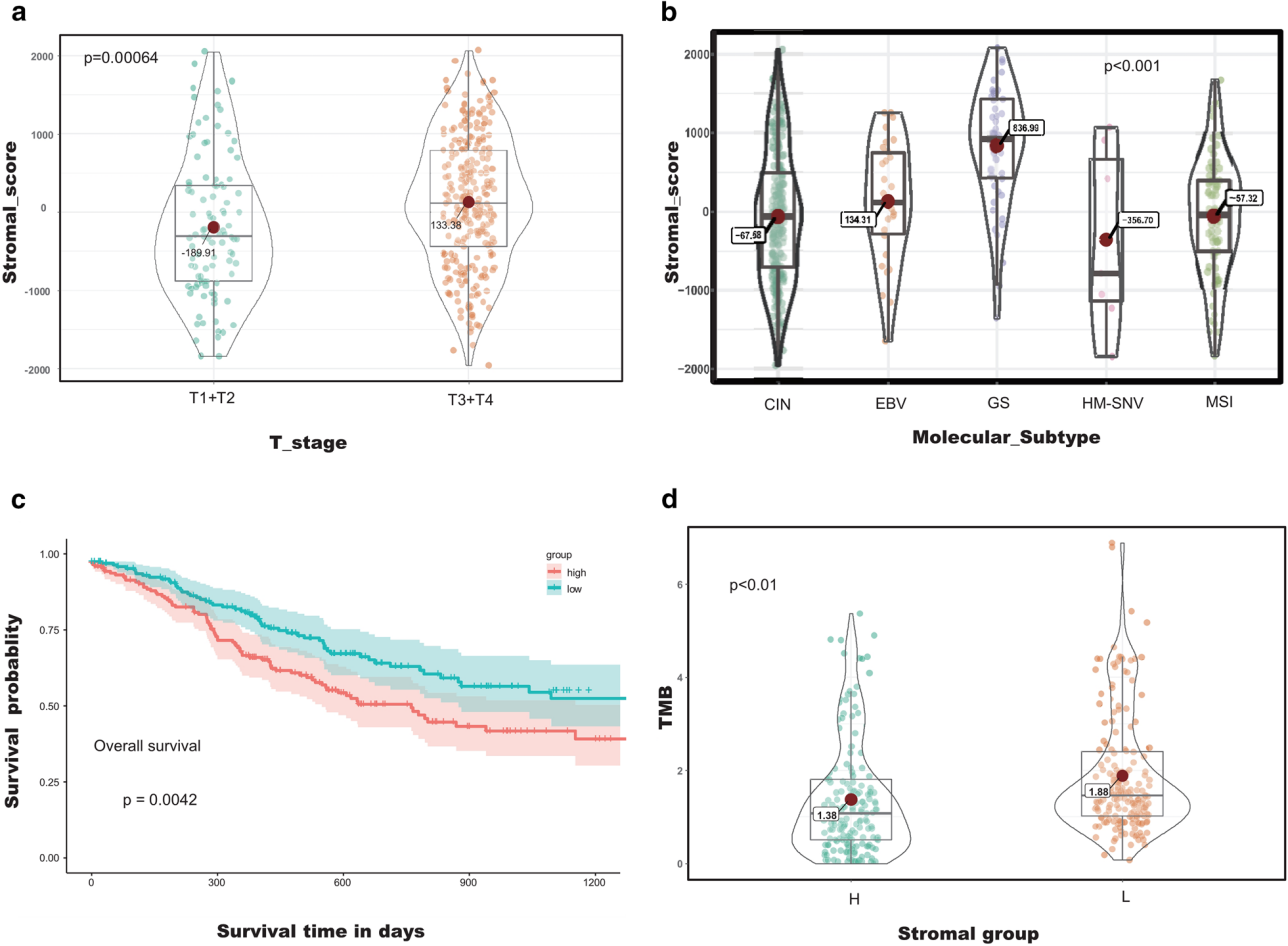
Stromal scores are associated with T-stage/overall survival/subtypes/TMB of gastric cancer. **a** Compared to the early T-stage, the advanced T-stage is associated with a higher stromal score (p < 0.001). **b** Distribution of stromal scores of GC subtypes. The Volin-Box-plot shows that there is a significant association between GC subtypes and the level of stromal scores (p < 0.001). **c** The TCGA GC cases were divided into the high and low stromal score groups by the optimal cutoff. Kaplan–Meier survival analysis shows that there is a statistically significant survival advantage for the low stromal score group (p = 0.0042); and **d** TMB for the different stromal score subtypes

Notably, the patients staged in the early T group averaged a lower stromal score than those in the advanced T group in both cohorts (Fig. 2a; Additional file 2: Figure S1) (*p* < 0.001 in TCGA cohort, *p* < 0.0001 in GEO cohort). According to the report of Peter et al. [32], GC can be divided into five subtypes (CIN, EBV, GS, HM-SNV, and MSI subtype) based on the molecular parameters of histology. Our results showed that the average stromal score of the GS subtype was the highest rank of all of the five subtypes, followed by that of EBV subtype, MSI subtype, CIN subtype, and HM-SNV subtype (Fig. 2b) (*p* < 0.001). To investigate whether stromal scores predict the outcome of GC patients, we divided all of the patients into two groups based on the optimal cutoff and performed Kaplan–Meier survival analysis. The results showed that patients with low stromal score have a survival advantage over those with a high stromal score (Fig. 2c) (*p* < 0.01). These results demonstrated that the stromal score is correlated with subtype classification as well as the outcomes of GC patients.

In addition, univariate and multivariate Cox regression analysis showed that the stromal score is an independent prognostic indicator of OS in patients from the TCGA database (Table 2) (*p* = 0.008). The GEO cohort further validated that the stromal score predicts the OS of GC patients using multivariate analysis (Additional file 3: Table S2) (*p* = 0.02).

**Table 2:** Univariate and multivariate analyses of stromal score and clinicopathological factors for OS in TCGA cases

Factor	Univariate analysis			Multivariate analysis		
	HR	p value	CI95	HR	p value	CI95
Age	0.67	0.021	0.48–0.94	0.53	0.001	0.37–0.77
Gender	1.26	0.204	0.88–1.82	1.35	0.133	0.91–1.99
T_stage	0.48	0.001	0.31–0.75	0.7	0.182	0.42–1.18
M_stage	0.4	0.001	0.23–0.7	0.39	0.003	0.21–0.72
N_stage	0.45	0	0.29–0.7	0.6	0.115	0.32–1.13
TNM stage	0.43	0	0.3–0.63	0.76	0.359	0.43–1.36
Stromal_group	0.66	0.015	0.47–0.92	0.62	0.008	0.43–0.88

### Identification of DEGs based on the status of stromal scores

**Fig. 3:**
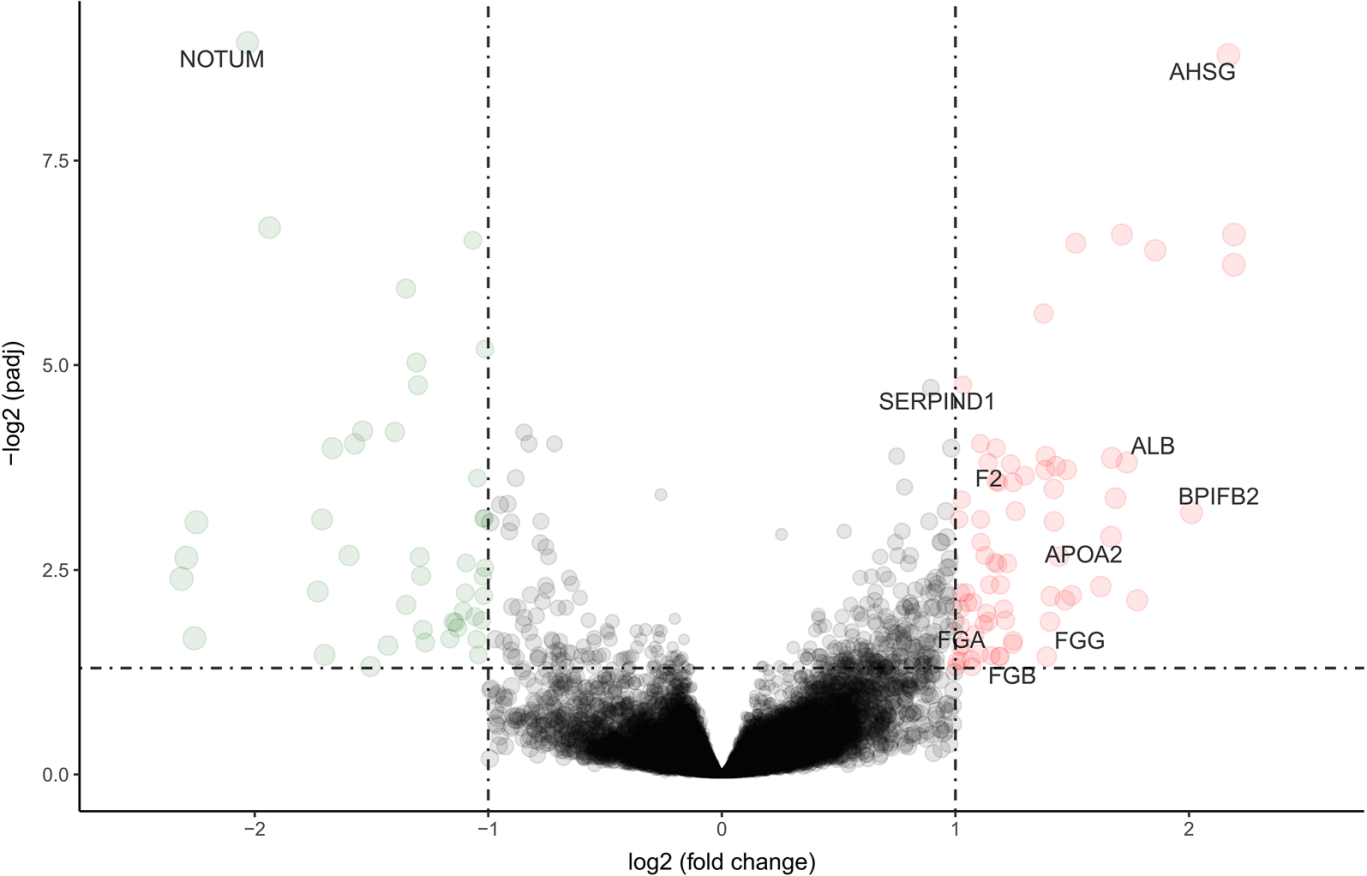
Volcano plot of the differentially expressed genes. Each dot represents a gene. Red dots indicate the upregulated genes. Green dots indicate downregulated genes. Black dots represent genes for which the differences are not significant

**Fig. 4:**
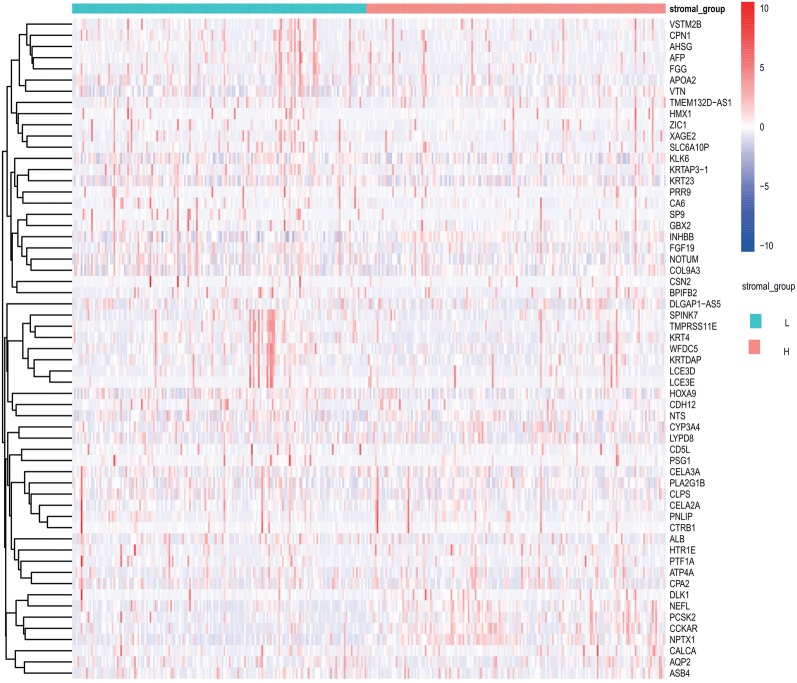
Heatmap of the representative DEGs of left half (low score) vs. right half (high score). The clustering of the DEGs is shown on the left, and gene symbols are shown on the right of the heatmap

By comparing the global gene expression of samples from the TCGA cohort with high versus low stromal scores, we identified 119 DEGs, including 74 upregulated genes and 45 downregulated genes (p < 0.05, log2 fold change (FC) > 1). Volcano plot (Fig. 3) and Heatmap (Fig. 4) showed the representatives of the DEGs.

### Functional enrichment analysis of the DEGs

**Fig. 5:**
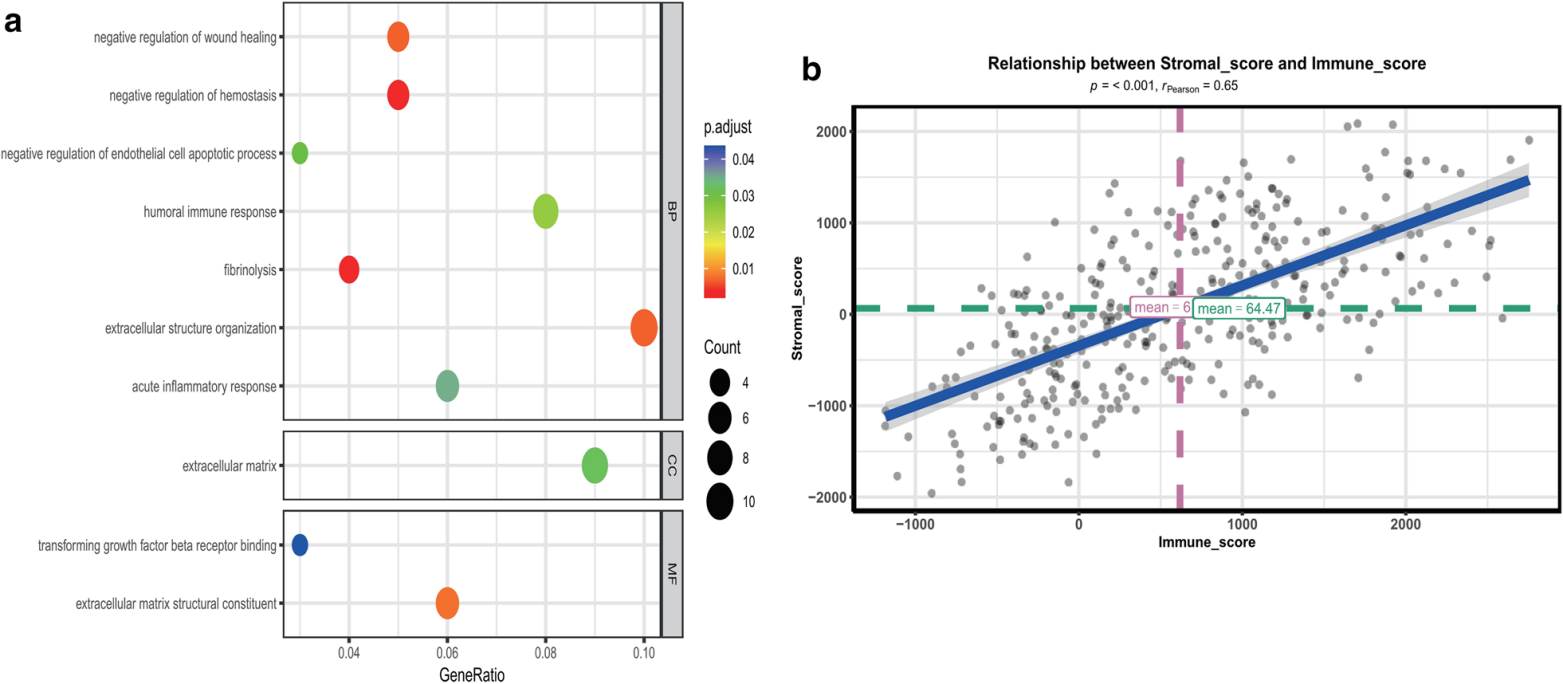
Functional enrichment analysis of DEGs and relation between the stromal and immune scores. **a** GO analysis of the DEGs. The x-axis indicates the gene ratio, and the y-axis indicates the GO terms. p-value is shown in different colors. The dot size represents the number of genes. **b** The stromal score is significantly positively correlated with the immune score (p < 0.001, R = 0.65)

The identified DEGs were subjected to GO analysis. For enriched biological processes (BP), DEGs were mainly enriched in negative regulation of wound healing, negative regulation of hemostasis, negative regulation of endothelial cell apoptotic process, humoral immune response, fibrinolysis, extracellular structure organization, and acute inflammatory response. DEGs in the molecular function MF category were mainly associated with transforming growth factor beta receptor binding, extracellular matrix structural constituent. Concerning the cell component (CC), DEGs primarily clustered in the extracellular matrix (Fig. 5a; Additional file 4: Table S3) (*p*.adjust < 0.05).

### The low stromal score group may be more sensitive to immunotherapies

**Fig. 6:**
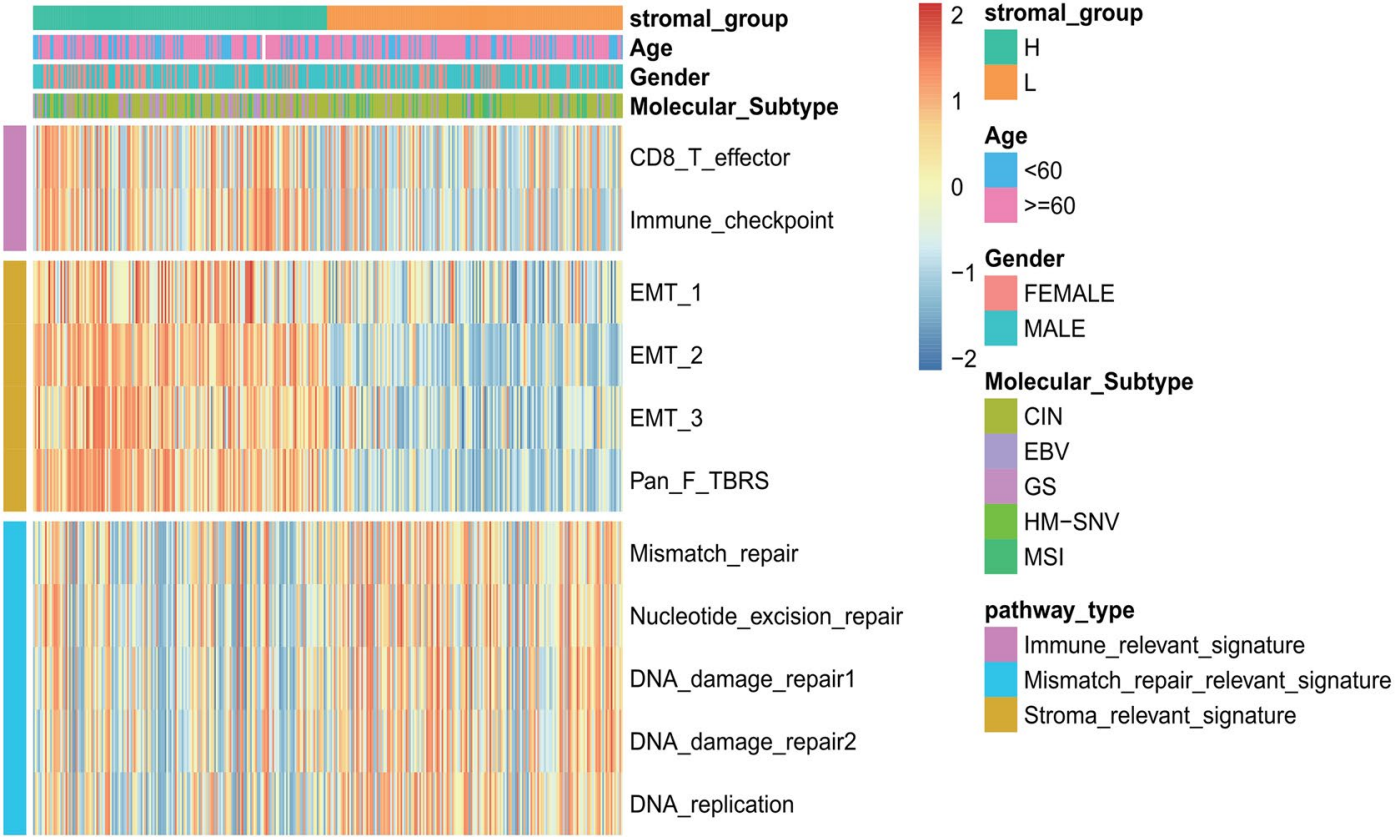
Characteristics of stromal subtype and functional annotation. The stromal subtypes are distinguished by different signatures (immune-relevant signature, mismatch-relevant signature, and stromal-relevant signature). Histological subtypes and the TME cluster are shown as patient annotations

As shown in Fig. 5b, there was a significantly positive correlation between the stromal score and the immune score (*p* < 0.001), demonstrating the crosstalk between the stromal cells and immune cells in the TME. Besides, the high stromal score group was associated with high expression of immune and stromal-relevant signatures, whereas the expression of MSI-related mRNAs was relatively low (Fig. 6). Furthermore, the low stromal score group may be more likely (62.6% in the TCGA cohort and 49.3% in the GEO cohort) to respond to immunotherapy than the high stromal score group (13.2% in the TCGA cohort and 23.3% in the GEO cohort) according to the TIDE algorithm. Interestingly, in good agreement with the TIDE algorithm, the low stromal score group presented a significantly higher count of TMB, which is significantly associated with the efficacy of immunotherapy (Fig. 2d) (*p* < 0.01).

Considering the associations between TMB, MSI relevant signatures, and immunotherapy, as well as results of TIDE algorithm, it can be referred that patients in the low stromal score group may be more sensitive to PD-1/PD-L1 checkpoint therapy than the high stromal score group patients. Interestingly, high stromal score group with high immunized terms was resistant to immunotherapy compared to low stromal score group, promoting stromal relevant pathways participate the process, consistent with studies [33–35], emphasizing that stromal activation is the core mechanism of resistance to checkpoint blockade.

### Survival analysis of DEGs

**Fig. 7:**
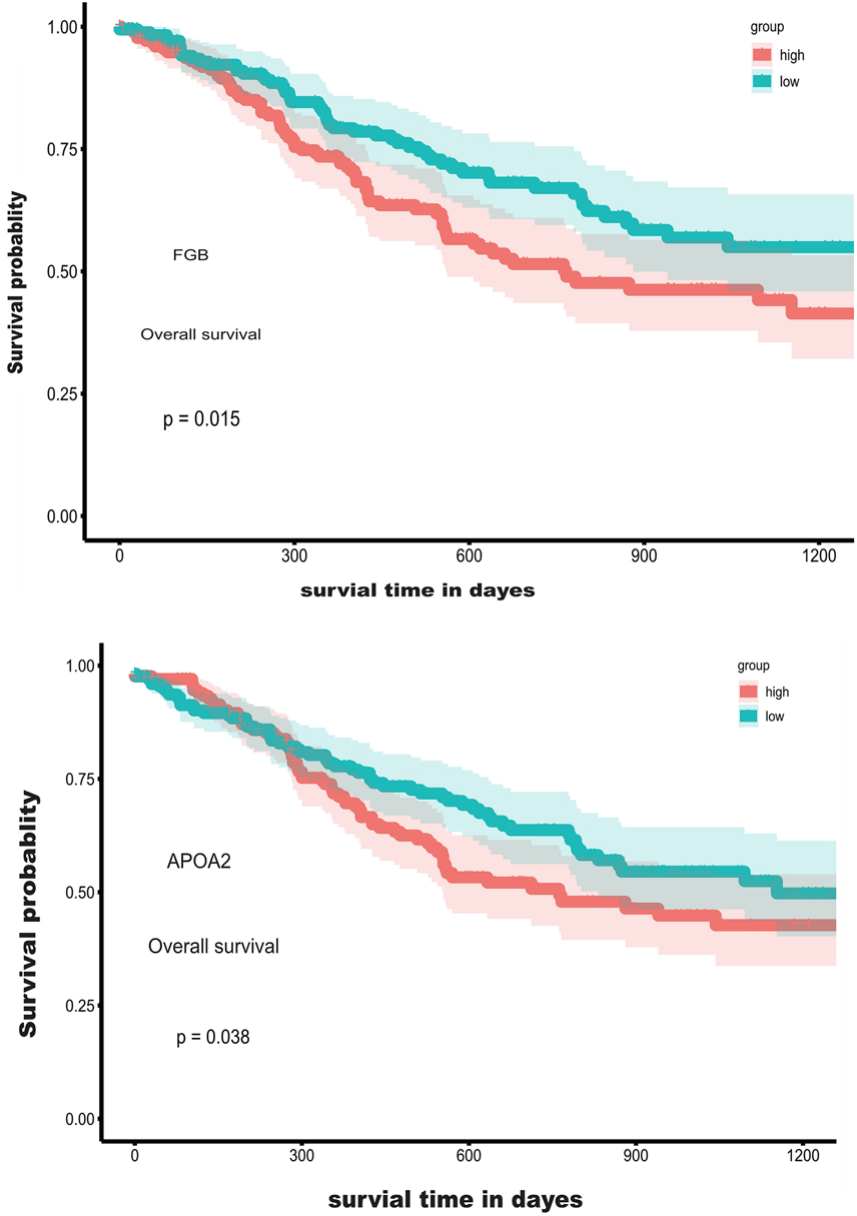
Correlation of expression of individual DEGs in the OS of patients from the TCGA database. Kaplan–Meier survival analysis was performed to compare OS between high (grey line) and low (black line) gene expression of selected DEGs

The Kaplan–Meier survival analysis with log-rank test was used to evaluate the potential roles of individual DEGs for OS. The results showed that seven upregulated and three down regulated DEGs predict poorer OS with statistical significance (Fig. 7; Additional file 5: Figure S2) (*p* < 0.05).

### Construction of protein–protein interaction (PPI) network

**Fig. 8:**
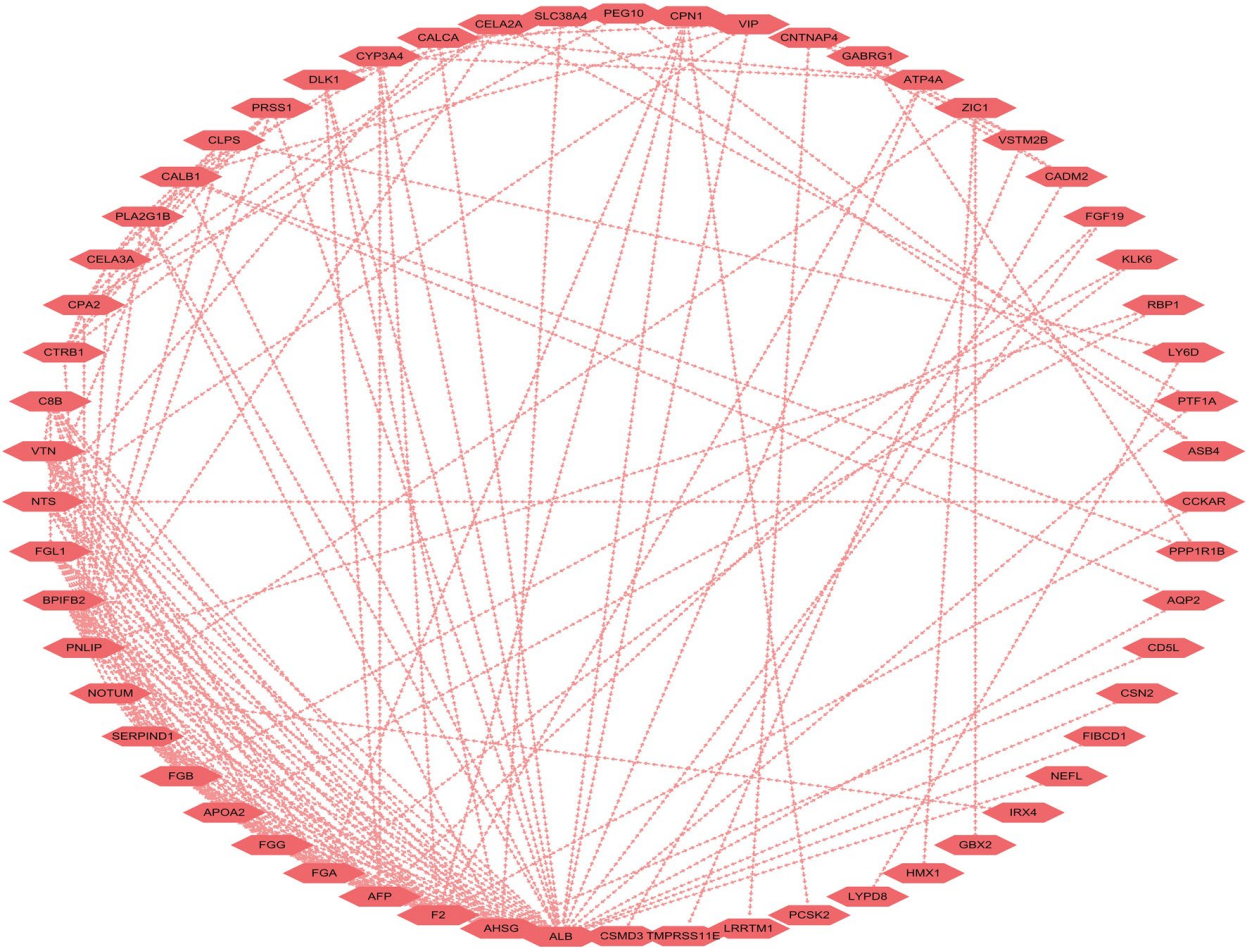
The protein-PPI network for the DEGs. Each node represents a gene. The dotted line indicates the relationship between the two genes. The number of lines is correlated with the degree scores of the genes

**Fig. 9:**
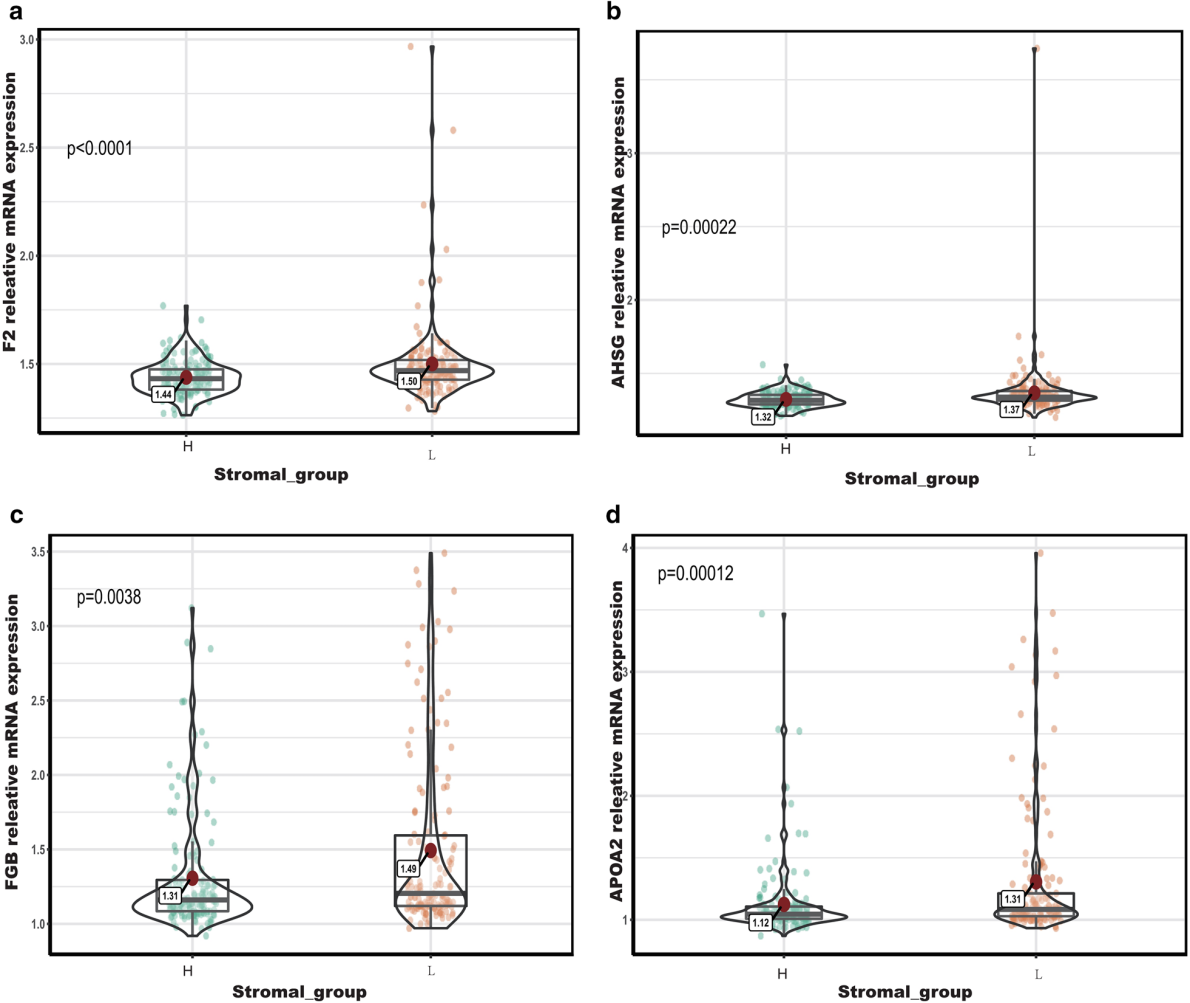
Validation of the hub genes in the GEO cohort. **a** F2: coagulation factor II; **b** AHSG: alpha 2-HS Glycoprotein; **c** FGB: fibrinogen beta chain; **d** APOA2: apolipoprotein A-II

To better understand the interplay among the identified DEGs, we obtained protein-PPI networks using STRING tool, and the pairs whose combined score > 0.4 were extracted for visualization by Cytoscape (Fig. 8). The 12 genes with the highest degree scores were selected, and the expression of these genes was validated in the GEO cohort (Fig. 9; Additional file 6: Figure S3). The common genes included F2, AHSG, AFP, FGA, FGB, APOA2, PNLIP, and BPIFB2. All of these genes had degree scores > 10 and were upregulated genes. These hub genes might contribute to stromal cell modulation in gastric cancer patients.

### GSEA and functional annotation of HUB genes

**Fig. 10:**
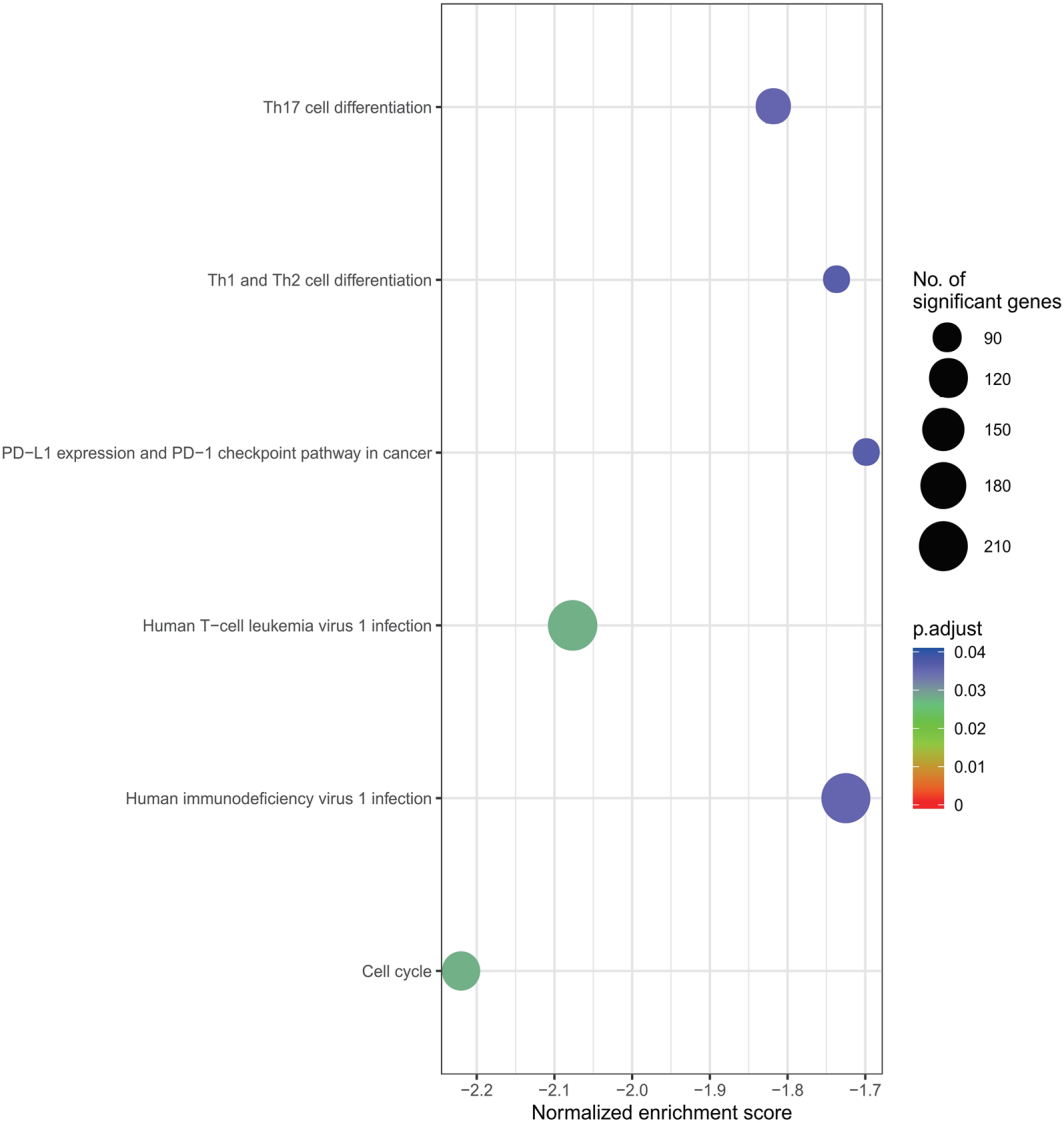
Enriched KEGG pathways of BPIFB2 by using GSEA. p-value is presented in different colors. The dot size represents the number of genes

To explore the potential function of hub genes in GC, we applied GSEA on the TCGA database using the KEGG gene sets. Interestingly, the most important enriched KEGG pathways of BPIFB2 included cell cycle, Human T-cell leukemia virus 1 infection, Th17 cell differentiation, human immunodeficiency virus 1 infection, Th1 and Th2 cell differentiation, PD-L1 expression and PD-1 checkpoint pathway in cancer (Fig. 10) (*p*.adjust < 0.05).

## Discussion

Immune-checkpoint inhibitors targeting PD-1 and PD-L1 have shown promising clinical results for GC [36–38]. The recent advances in the relationship between gene expression and TME in gastric cancer patients [39–41] highlight the link between the immune-cell infiltration and PD-1/PD-L1 expression. However, only a subset of patients that contain tumor-infiltrating immune cells respond to PD-1/PD-L1 inhibitors. The pivotal role of the stroma is now receiving wide attention. Emerging data suggest that targeting CAFs/anti-angiogenic therapy in combination with anti-PD-1 immunotherapy is an effective way to overcome GC [20, 42].

In this study, we investigated a study cohort of 383 gastric cancer patients in the TCGA database and 300 patients in the GEO cohort, and generated stromal scores using the ESTIMATE algorithm. Our results showed that the stromal score is a robust biomarker for predicting survival in GC and guiding more effective immunotherapy strategies. Patients in the low stromal score group have a survival advantage over those in the high stromal score group and would benefit more from anti-PD-1 therapy. Interestingly, this result is consistent with the finding that GC tumors classified as MSI, which is a relatively low stromal score subtype, show promising results for the use of PD-1/PD-L1 blockade [38–40]. In line with previous research [43], the high stromal score subtype showed activation of transforming growth factor and epithelial mesenchymal, which were considered T-cell suppressive [33–35] and to be involved with immunotherapy resistance.

We then identified 119 DEGs, and many of the DEGs were involved in immune and stromal modulation. Of these 119 DEGs, ten genes were found to be associated with outcomes in GC patients. F2, AHSG, AFP, FGA, FGB, APOA2, PNLIP, and BPIFB2 were selected as the hub genes. BPIFB2 was involved with PD-L1 expression and PD-1 checkpoint pathway in cancer and immune-cell modulation. To date, limited data are available on BPIFB2. At the ESMO Asia 2018 Congress, Z. Karim and colleagues showed that BPIFB2 leads to gene expression alteration in the EMT markers. Together, these results suggest that BPIFB2 may be an effective treatment for PD-1/PD-L1 resistance.

Of these hub genes, FGB and APOA2 were identified to predict poorer survival in this cohort. More recently, Moriggi et al. reported that fibrinogen, encoded by FGB, is involved in ECM remodeling, including the epithelial–mesenchymal transition (EMT) [44]. Matthew D. Galsky further confirmed that EMT-related gene expression and T-cell infiltration are positively correlated, and the balance of T-cell vs. EMT/stromal elements may have prognostic/predictive implications with the antitumor immune response [45].

We were also interested in other hub genes that may play an essential role in tumor stromal cell infiltration, although they are not associated with patients’ survival. F2, which encodes the coagulation factor II (also known as thrombin), is a pro-inflammatory and pro-coagulant molecule that is elevated in various cancers, including breast and gastric cancers [46, 47]. Recent studies showed that disorders of the coagulation-fibrinolysis system are associated with the efficacy of immune-checkpoint inhibitors [48]. Thrombin activates platelets in the tumor microenvironment and induces angiogenesis by various mechanisms, including the section of pro-angiogenic factors and the promotion of the EMT [49–51]. AHSG encodes fetuin-A (also known as alpha 2-HS Glycoprotein), a 59-kDa negative acute phase glycoprotein in humans that is predominantly synthesized by liver parenchymal cells [52]. Recent studies indicated that fetuin-A mediates the attachment and growth of tumor cells in an indirect way, which involves cellular exosomes [53]. Fetuin-A is also associated with dendritic cell and T-regulatory cells [54].

## Conclusion

Conclusively, the evaluation of the stromal component in TME, and molecular, genetic factors associated with TME stromal infiltration, revealed that the stromal score may be a biomarker to predict response to immune checkpoint and prognosis of GC patients. The related genes and signal pathways, provide a new idea for novel drug combination strategies of GC. Further investigation should be performed to confirm the potential genes and signaling pathways.

## Supplementary information

**Additional file 1: Table S1.** Association between stromal score expression and the clinical parameters in the GEO cohort.

**Additional file 2: Figure S1.** Stromal scores were associated with T-stage in the GEO cohort. A high stromal score is associated with advanced T-stage.

**Additional file 3: Table S2.** Univariable and multivariable analyses of the stromal score and clinical variables in the GEO cohort.

**Additional file 4: Table S3.** Go terms of DEGs.

**Additional file 5: Figure S2.** Kaplan–Meier survival analysis of C8B, GFL1, INHBB, PRSS1, RHOXF2, SLC38A4, VTN, and Z1C1.

**Additional file 6: Figure S3.** Comparison of AFP, FGA, PNLIP, and BPIFB2 in different groups of the stromal score.

## Data Availability

The data that support the findings of this study are available from TCGA and GEO databases.

## Abbreviations

PD-1: Programmed cell death 1
PD-L1: Programmed death-ligand 1
GC: Gastric cancer
GEO: Gene Expression Omnibus
GSEA: Gene set enrichment analysis
OS: Overall survival
TME: Tumor microenvironment
CAF: Cancer-associated fibroblasts
ESTIMATE: Estimation of stromal and immune cells in malignant tumor tissues using expression data
TCGA: The Cancer Genome Atlas
DEGs: Deferentially expressed genes
TMB: Tumor mutation burden
GO: Gene ontology
PPI: Protein–protein interaction
EMT: Epithelial–mesenchymal transition
