# Supplementary material for: Identification and validation of stromal-tumor microenvironment-based subtypes tightly associated with PD-1/PD-L1 immunotherapy and outcomes in patients with gastric cancer
Source: Cancer Cell Int. 2020 Mar 24;20:92. doi: 10.1186/s12935-020-01173-3 (PMC7092673; doi:10.1186/s12935-020-01173-3)
Supplement: Supplementary file 1 — Additional file 1: Table S1. Association between stromal score expression and the clinical parameters in the GEO cohort. [file 12935_2020_1173_MOESM1_ESM.docx]

Additional file 1: Table S1. Association between stromal score expression and the clinical parameters in the GEO cohort.
