# Supplementary figures and images for: Identification and validation of stromal-tumor microenvironment-based subtypes tightly associated with PD-1/PD-L1 immunotherapy and outcomes in patients with gastric cancer
Source: Cancer Cell Int. 2020 Mar 24;20:92. doi: 10.1186/s12935-020-01173-3 (PMC7092673; doi:10.1186/s12935-020-01173-3)

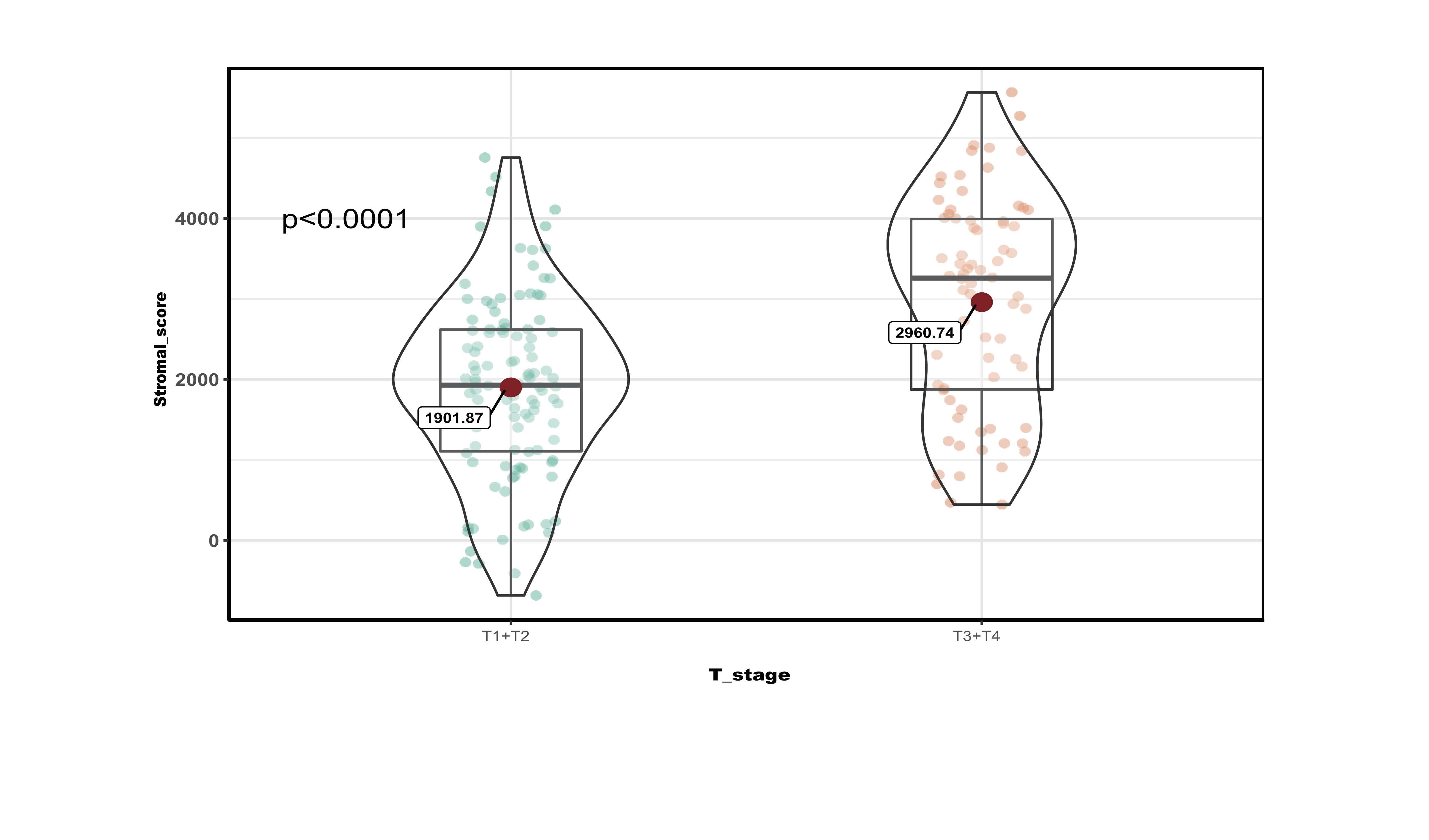

Supplement: Supplementary file 2 — Additional file 2: Figure S1. Stromal scores were associated with T-stage in the GEO cohort. A high stromal score is associated with advanced T-stage. [file 12935_2020_1173_MOESM2_ESM.jpeg]

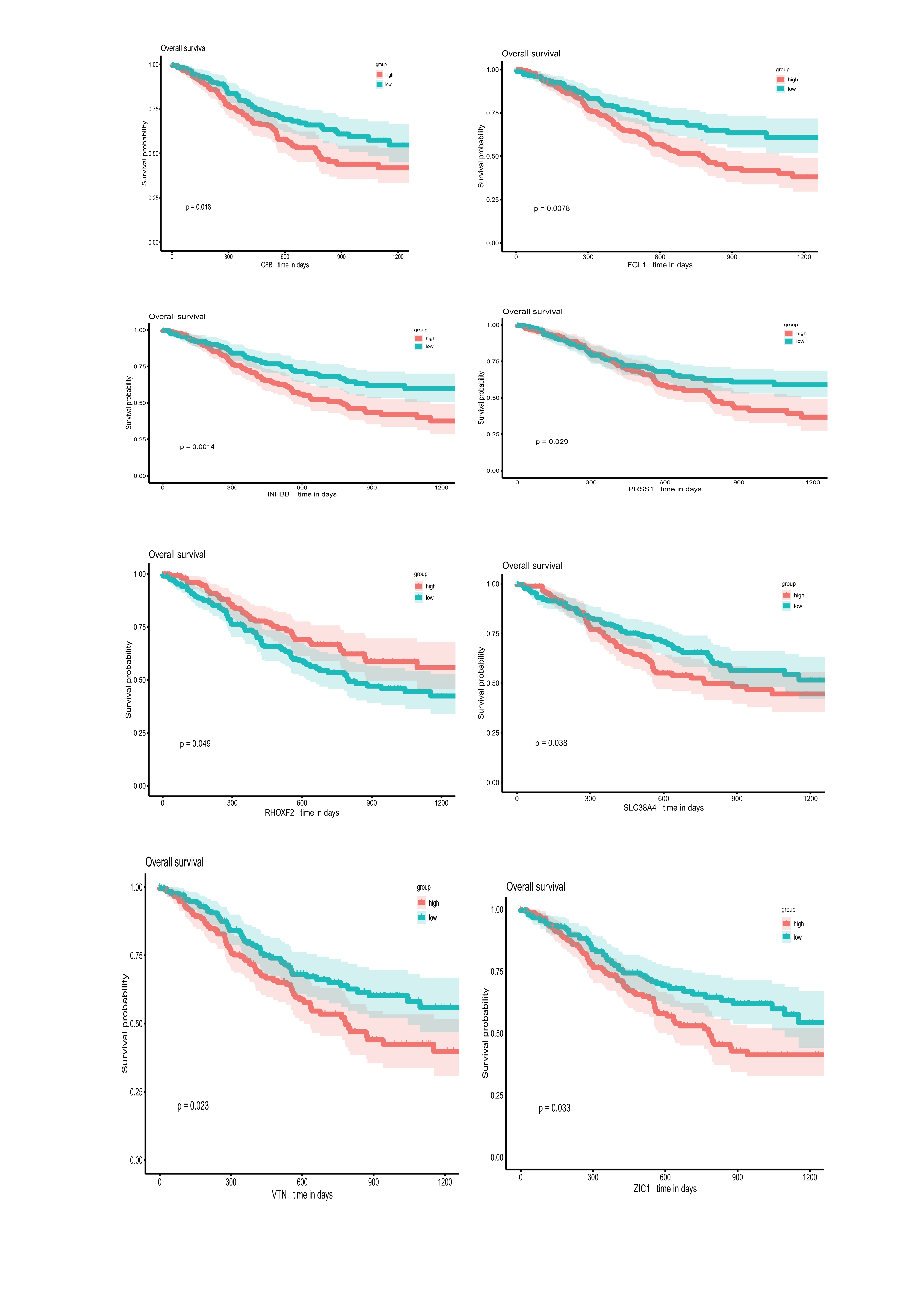

Supplement: Supplementary file 5 — Additional file 5: Figure S2. Kaplan–Meier survival analysis of C8B, GFL1, INHBB, PRSS1, RHOXF2, SLC38A4, VTN, and Z1C1. [file 12935_2020_1173_MOESM5_ESM.jpeg]

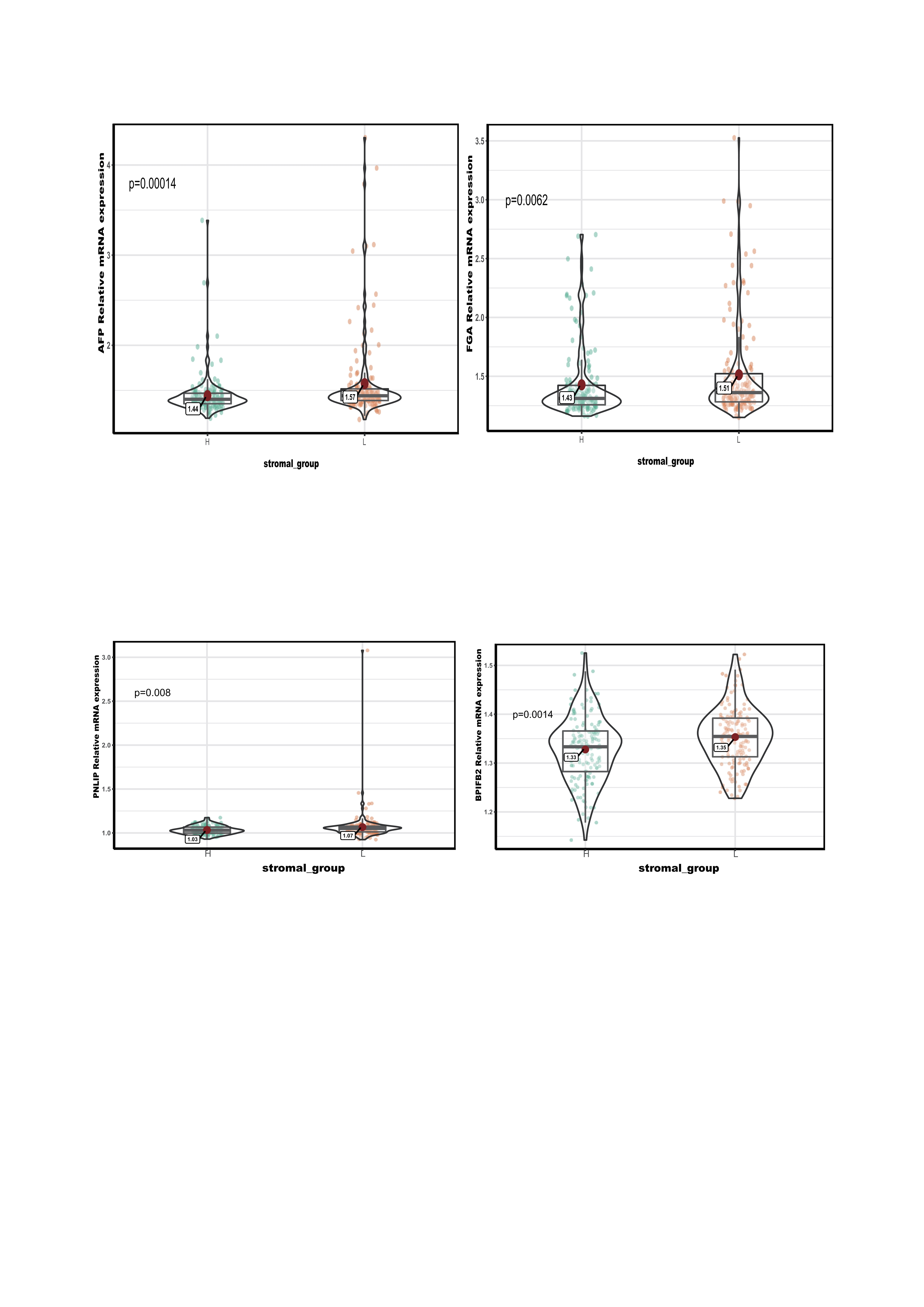

Supplement: Supplementary file 6 — Additional file 6: Figure S3. Comparison of AFP, FGA, PNLIP, and BPIFB2 in different groups of the stromal score. [file 12935_2020_1173_MOESM6_ESM.jpeg]
