# Supplementary material for: Identification and validation of stromal-tumor microenvironment-based subtypes tightly associated with PD-1/PD-L1 immunotherapy and outcomes in patients with gastric cancer
Source: Cancer Cell Int. 2020 Mar 24;20:92. doi: 10.1186/s12935-020-01173-3 (PMC7092673; doi:10.1186/s12935-020-01173-3)
Supplement: Supplementary file 4 — Additional file 4: Table S3. Go terms of DEGs. [file 12935_2020_1173_MOESM4_ESM.docx]

Additional file 4: Table S3. Go terms of DEGs
